# Moving through menopause: a mixed methods study of UK women’s experiences of being physically active during the menopause life stage

**DOI:** 10.1097/GME.0000000000002641

**Published:** 2025-11-04

**Authors:** Ailsa G. Niven, Tessa Strain, Janis Reid, Divya Sivaramakrishnan, Nanette Mutrie, Claire Fitzsimons

**Affiliations:** 1 Physical Activity for Health Research Centre, Institute Sport, Physical Education and Health Sciences, Moray House School of Education and Sport, University of Edinburgh, Holyrood Road, Edinburgh, UK; 2 Scottish Collaboration for Public Health Research and Policy (SCPHRP) School of Health in Social Science University of Edinburgh, Scotland, UK

**Keywords:** Behavior change theory, Menopause, Mixed methods, Physical activity, Women’s health

## Abstract

**Objective:**

There is growing evidence for the benefits of physical activity during the menopause life stage. However, limited research has explored physical activity behavior of UK women. Using a mixed-methods approach, 2 phases of research were undertaken to: (1) assess physical activity levels and examine the relationship with menopausal symptoms, and (2) use the COM-B theoretical framework to understand the influence of “Capability, Opportunity and Motivation” on physical activity Behavior.

**Methods:**

In phase 1, participants completed an online survey (n = 655; mean age = 49.9 y). Descriptive analyses were supplemented with χ^2^ tests, with Bonferroni correction. In phase 2, 4 online focus groups (n = 24; mean age = 52.7 y) were undertaken and thematically analyzed.

**Results:**

In phase 1, 75% reported achieving 150 minutes of moderate to vigorous physical activity/week, although 57% reported activity levels had decreased during the menopause life stage. Twelve out of 14 symptoms were experienced by >50%, with changes in mood and brain fog most common (>80%). There was no significant difference in the proportion meeting the moderate to vigorous physical activity guidelines between those women who did or did not experience individual symptoms, although for 10/14 symptoms, >50% indicated a negative impact on likelihood to engage in activity. In phase 2, capability (eg, menopausal symptoms), opportunity (eg, social support), and motivation (eg, low confidence) were all influential on behavior.

**Conclusion:**

These findings enhance our understanding of UK women’s experiences of being physically active during the menopause, and provide insight into potential intervention strategies to support women to be active at that time.

Natural menopause is clinically diagnosed at 12 months after the final menstrual period resulting from the loss of ovarian follicular activity.^1^ The average age of menopause in the UK is 51,^2^ although there is cultural variation.^3^ The menopause transition (time leading up to the menopause) and postmenopause stages of reproductive aging are also critical phases in this reproductive aging process,^1,4^ and we use the term “menopause life stage” as an inclusive term. Symptoms associated with menopause are “likely” to occur in the late menopause transition phase and “most likely” to occur in the first 2 years of the early postmenopause stage.^1^ A wide range of symptoms are associated with these stages of the menopause^5^ including physical (eg, vasomotor, body aches and pains, reduced libido) and mental health (eg, nervousness and anxiety; problems with memory and concentration) symptoms.^2^ The British Menopause Society (BMS) reports that more than 75% of women experience menopausal symptoms, with over 25% describing severe symptoms, and an average duration of symptoms of 7 years.^6^

Given the range and prevalence of menopausal symptoms, effectively managing these symptoms is essential for women’s well-being. Many women seek medical support;^7^ although the accessibility and quality of support provision can vary.^8^ A recent review^9^ on the management of common menopausal symptoms, including vasomotor, mood and cognitive changes, sleep disorders and bone loss, emphasized that hormonal therapies are the most effective treatment for many symptoms. Although other treatments were also considered, there was limited consideration of the alternative or adjunct benefits of lifestyle interventions. Nevertheless, the importance of lifestyle advice is highlighted in NICE guidelines,^10^ and recent UK national strategies for women’s health.^11,12^ Physical activity is one such lifestyle behavior.

There is substantial evidence for the health benefits of meeting recommended levels of physical activity^13^ across the lifespan.^14^,^15^,^16^ Furthermore, there is now a growing evidence base for the benefits of physical activity during the menopause life stage. Specifically, review-level evidence supports the benefits for general health and well-being,^17^,^18^,^19^,^20^ managing menopausal symptoms,^20^,^21^,^22^,^23^ and as a protective strategy for longer-term morbidity (eg, osteoporosis^24^). Despite the growing evidence, there is a recurring theme of low numbers of studies, imprecise defining of the menopause life stage, and poor methodological quality within the evidence base, highlighting the need for further research.

Although there are substantial benefits of physical activity for health and well-being, women are consistently less physically active than men^25^ and decreases in physical activity levels are evident in midlife.^26,27^ While the determinants of physical activity behavior are multifactorial,^28^ there is a need to better understand how the menopause life stage impacts women’s experiences to best support them to be physically active.

A recent systematic review^29^ adopting the social-ecological framework highlighted that a range of factors impacted physical activity behavior during the menopause, with intrapersonal (eg, menopause symptoms) being particularly prominent. Several cross-sectional observational studies have also indicated an inverse relationship between menopausal symptoms and physical activity levels.^18,30,31^ While such studies add to our understanding, due to the nature of these studies, it is not possible to untangle the direction of effect (ie, does being more active result in fewer symptoms, or are women with fewer symptoms more likely to be active). Qualitative studies have the potential to provide a deeper insight into the experiences of women. However, there is a paucity of such research to date, with most studies focusing on understanding women’s experiences of specific physical activity interventions^32^,^33^,^34^ or types of activity,^35,36^ or focused on specific groups (eg, Black women^37^), or of small scale (eg, n = 9;^38^ n = 4^39^).

McArthur et al^40^ interviewed 53 women who were experiencing menopausal symptoms and identified a range of barriers (eg, competing demands) and enablers (eg, expected benefits) to being physically active. However, this study and other qualitative studies have typically not utilized theoretical frameworks that can be valuable to effectively inform future interventions designed to support women to be more active. One theoretical framework is the Behavior Change Wheel (BCW),^41^ which has been used effectively in physical activity research,^42,43^ including menopause-related research.^44^ In brief, the COM-B component of the BCW model provides a framework to understand the influence of “capability” (psychological and physical), “opportunity” (social and physical), and “motivation” (automatic and reflective) on “behavior” to identify where to target interventions.

The overall aim of this research was to increase understanding of the intersection of the menopause and physical activity in a sample of UK-based women to inform future support. There is considerable value in developing a country-specific understanding of the relationship between PA and menopause life stage due to the cultural variation in the average age of menopause and prevalence of symptoms, as well as variation in opportunities for medical support and physical activity by country. Using a mixed-method approach, 2 phases of research were undertaken to address 2 objectives:

- To assess physical activity levels and examine the relationship with menopausal symptoms.
- To capture women’s lived experiences and use the COM-B framework to understand the influence of capability, opportunity and motivation on physical activity behavior during the menopause life stage.

## PHASE 1: SURVEY

## METHOD

### Participants

Women in the UK who self-assessed as being perimenopausal, early postmenopause, or mid postmenopause of a naturally occurring menopause (ie, not chemically or surgically induced, nor due to premature ovarian insufficiency) were invited to participate in the study through several recruitment routes. These routes included social media posts (eg, X and Facebook) and email newsletter communications by the researchers and a range of partners representing both physical activity and wider health networks. These partners included a mental health charity (and research funder; SAMH), Public Health Scotland and a range of physical activity organizations (eg, JogScotland), and the Active Scotland Division of The Scottish Government. We initially targeted women in Scotland due to the focus of the funder, but the use of social media resulted in participants from across the UK being recruited, and we opted to include all participants.

### Instrument

An online questionnaire with 8 sections was developed using Qualtrics^TM^ (Provo, UT). Two sections on mental well-being will be reported elsewhere.

#### Demographic characteristics

Questions on age, ethnicity, gender identity, and socioeconomic status (as assessed by postcode and converted using Scottish Index of Multiple Deprivation; SIMD^45^) were included.

#### Menopausal status

Current menopausal status was self-assessed based on the STRAW-10 classification.^1^ Participants indicated if they were perimenopausal (ie, last menstrual period was <12 months ago, but menstrual periods are irregular), early postmenopause (ie, up to 2 y since last menstrual period) or mid postmenopause (ie, 2 to 8 y since last menstrual period).

#### Physical activity behavior

The Scot-PASQ^46^ was used to assess whether participants met the current UK Chief Medical Officers (CMO) recommended levels of 150 minutes of moderate to vigorous physical activity (MVPA).^13^ In line with the current CMO recommendations, we also created a question to assess the prevalence of achieving the recommendation to undertake muscle strengthening activity at least twice a week (ie, “*In the last week, have you completed an activity on* *2 or more occasions that made your muscles feel some tension, shake or feel warm?*”). Finally, participants were asked if their PA levels had increased or decreased during the current menopause lifestage, and if they were interested in becoming more active.

#### Menopausal symptoms

Participants were presented with a range of menopausal symptoms as outlined on the UK National Health Service (NHS) website,^47^ with one additional symptom relating to difficulty sleeping (but not due to night sweats), and asked to indicate if they had or had not experienced each symptom (ie, responded “yes” or “no”).

#### Impact of menopausal symptoms on physical activity

Participants were asked to indicate the extent to which each menopausal symptom experienced impacted on their likelihood to engage in physical activity on a 5-point scale from “a lot less likely” to a “lot more likely” to engage. If participants did not experience individual symptoms, they could report “not applicable,” and they were excluded from the analysis of this question.

### Procedure

After institutional sponsorship and ethical approval (Ref: NAIL02082022), from October 12, 2022 to November 7, 2022, eligible participants were invited to complete the online questionnaire. To access the questionnaire, participants were required to complete an online consent form. Given the sensitivity of the topic, there were no forced-response questions, with the exception of the question relating to menopausal status. For the data to be included, participants were required to actively “submit” their data at the end of the questionnaire as an additional safeguard to reconfirm consent. Participants were incentivized to participate with the optional opportunity to be entered into a prize draw for one of five £50 vouchers. Participation was anonymous and personal data were only collected if participants provided their email address to enter the prize draw, or to indicate interest in further research. These personal data were disaggregated from the research data at the first opportunity.

### Data cleaning and analysis

Before analysis, we excluded participants who had not formally “submitted” their response, participants who were aged 35 or under, as they could not be reliably classified as experiencing premature menopause, and any duplicate responses (manually identified through identical free-text responses in subsequent lines of the data). Some participants did not respond to all questions, resulting in variation in participant numbers for different questions.

Descriptive analyses were undertaken to report on demographic characteristics, menopausal status, physical activity levels, prevalence of menopausal symptoms, and extent to which participants perceived symptoms impacted on their physical activity levels. We used χ^2^ tests to compare the proportions meeting the MVPA guideline by experience of each menopausal symptom. As the statistical analysis for the whole project involved 74 separate hypothesis tests, we used a Bonferroni corrected alpha level of 0.000675 (0.05/74). While this controls for type 1 errors (false positives), it increases the risk of type 2 errors (false negatives). Where relevant, we present CIs to assist in the interpretation of the data.

## RESULTS

### Demographic characteristics

Participants who had not formally “submitted” their response (n = 407), participants who were aged 35 or under (n = 95), and duplicate responses (n = 72) were excluded from analysis. This provided a data set of 655 responses, and Table 1 provides a summary of demographic characteristics for the included participants. The average age was 49.9 years and ranged from 36 to 67, with 44.3% <50 years, 51.7% between 50 and 59 years and only 4.0% 60 years or over. The majority of participants were White (96.2%), with a gender identity that matched the sex assigned at birth (99.1%). Based on reported postcode, we identified participants’ Scottish Index of Multiple Deprivation (SIMD) classification as an indicator of socioeconomic status. Nearly 25% provided a postcode that was not found in the SIMD database, potentially because the participants lived outside Scotland. From available data, there was representation of socioeconomic status across all 5 categories, but with a skew towards areas of more affluence. More than half the sample (56%) identified as perimenopausal, with 20% early postmenopause and 24% mid postmenopause. Just over half of the sample reported that they were currently taking hormone therapy.

**TABLE 1:** Demographic characteristics of participants

Demographic characteristic	n (%)^*a*^
Age (y)	
<50	278 (44.3)
50-59	324 (51.7)
≥60	25 (4.0)
Ethnicity	
White	630 (96.2)
Asian, Scottish Asian, British Asian	14 (2.1)
African, Scottish African, British African	9 (1.4)
Caribbean or Black	1 (0.2)
Prefer not to say	1 (0.2)
Gender identity same as sex at birth	
Yes	631 (99.1)
No	4 (0.6)
Prefer not to say	2 (0.3)
Socioeconomic status (SIMD quintiles)	
1 most deprived	39 (7.9)
2	77 (15.7)
3	88 (17.9)
4	138 (28.0)
5 least deprived	150 (30.5)
Menopausal status	
Perimenopause	367 (56.0)
Early postmenopause	131 (20.0)
Mid postmenopause	157 (24.0)
Currently taking any form of prescribed hormone therapy	
Yes	337 (51.5)
No	317 (48.5)

SIMD, Scottish Index of Multiple Deprivation.

^*a*^ Percentage of all valid responses.

### Physical activity behavior during the menopause life stage

Based on the responses to the Scot-PASQ, 75% met the current CMO recommended levels of 150 minutes of moderate to vigorous physical activity (MVPA) each week. In terms of muscle strengthening activity, 54.1% reported engaging in a form of physical activity that made their muscles feel tense, shake or warm on 2 or more times each week. Of participants, 48% achieved both the 150 minutes of MVPA and twice-weekly strength training recommendations. In terms of changes in physical activity levels during the current menopause life stage, 16.4% reported that their levels had increased, 23.4% reported stayed the same, 57.2% reported that their physical activity levels had decreased, and 74.6% indicated that they were interested in becoming more active.

### Menopausal symptoms and relationship with physical activity

Table 2 illustrates percentage of participants who had experienced each menopausal symptom, ordered from the highest proportion of “yes” responses to the lowest. Table 3 shows the difference in proportions meeting the MVPA guidelines among those who did and did not experience each symptom. None of the comparisons reached the adjusted threshold for statistical significance. The largest percentage point (pp) differences were observed for the symptoms of “muscle aches and joint pains,” “difficulty sleeping (not due to night sweats),” and “brain fog” where the proportion meeting the guidelines was 11.6 pp (95% CI: 4.4 to 18.8), 10.6 pp (95% CI: 3.2 to 18.0), and 9.6 pp (95% CI: 0.2 to 19.1) higher among those not experiencing the symptom, respectively. Meanwhile, the proportion meeting the guidelines was 9.5 pp (95% CI: −16.6 to −2.3) higher among those who experienced “difficulty sleeping (due to night sweats)” compared with those who did not.

**TABLE 2:** Percentage of the sample experiencing each menopausal symptom

Menopausal symptom	%
Change to mood (low mood, anxiety, mood swings, low self-esteem)	93.8
Brain fog	89.4
Changed body shape and weight gain	79.9
Muscle aches and joint pains	77.9
Difficulty sleeping (not due to night sweats)	76.9
Reduced sex drive	75.2
Hot flushes	71.0
Skin changes include dry and itchy skin	70.6
Difficulty sleeping due to night sweats	63.8
Palpitations	62.9
Headaches and migraines worse than usual	54.1
Vaginal dryness and pain	52.5
Heavy and prolonged periods	41.8
Recurrent UTI	24.0

UTI, urinary tract infection.

**TABLE 3:** Comparison between those experiencing or not experiencing each menopausal symptom on the proportion of participants meeting the MVPA guidelines

	n (%) meeting MVPA guidelines			
Symptom	Experiencing symptom	Not experiencing symptom	Percentage point difference in the proportion meeting MVPA guidelines between those not experiencing symptom and those who do (95% CI)	*P*
Changes to mood (low mood, anxiety, mood swings, low self-esteem)	447 (74.9)	33 (82.5)	7.6 (−4.7 to 19.9)	0.279
Brain fog	423 (74.2)	57 (83.8)	9.6 (0.2 to 19.1)	0.083
Changed body shape and weight gain	372 (74.0)	100 (78.7)	4.8 (−3.3 to 12.9)	0.266
Muscle aches and joint pains	356 (72.7)	118 (84.3)	11.6 (4.4 to 18.8)	0.005
Reduced sex drive	352 (74.4)	122 (78.2)	3.8 (−3.8 to 11.4)	0.341
Difficulty sleeping (not due to night sweats)	340 (72.5)	118 (83.1)	10.6 (3.2 to 18.0)	0.011
Hot flushes	333 (74.7)	140 (76.1)	1.4 (−5.9 to 8.8)	0.707
Skin changes	325 (73.5)	146 (78.9)	5.4 (−1.8 to 12.6)	0.155
Difficulty sleeping (due to night sweats)	317 (79.1)	160 (69.6)	−9.5 (−16.6 to −2.3)	0.008
Palpitations	297 (74.8)	178 (76.1)	1.3 (−5.7 to 8.2)	0.724
Vaginal dryness and pain	245 (75.4)	223 (75.3)	0.0 (−6.8 to 6.7)	0.989
Headaches and migraines worse than usual	239 (71.6)	228 (80.0)	8.4 (1.7 to 15.1)	0.015
Heavy and prolonged periods	192 (75.0)	268 (74.4)	−0.6 (−7.5 to 6.4)	0.876
Recurrent UTI	115 (78.8)	341 (73.7)	−5.1 (−12.9 to 2.6)	0.214

MVPA, moderate to vigorous physical activity; UTI, urinary tract infection.

**FIG. 1:**
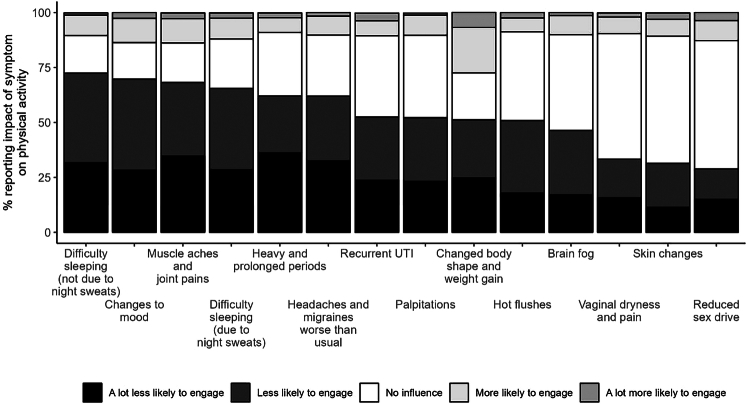
Impact of menopausal symptoms on likelihood to engage in physical activity. UTI, urinary tract infection.

Figure 1 illustrates the extent to which participants reported each symptom either positively or negatively impacted on their likelihood to engage in physical activity, with findings ordered from left to right by the highest to lowest proportion of “a lot less likely to engage” responses. For 10 out of the 14 symptoms, more than 50% of respondents indicated it had a negative impact on their likelihood to engage in physical activity. Difficulty sleeping (not due to night sweats), changes to mood, and muscle aches and joint pains had the most detrimental impact with around 70% of respondents indicating these symptoms resulted in them being a lot less likely or less likely to engage in physical activity.

## PHASE 2: FOCUS GROUP

## METHOD

### Participants

Participants were recruited from the survey respondents who had consented to being contacted regarding follow-up research, provided a valid email address, and agreed to participate.

### Instruments

Menopause status and demographic characteristics: An online questionnaire with 2 sections was developed using Qualtrics^TM^ (Provo, UT).   As in phase 1, participants self-identified if they were in the perimenopause, early postmenopause or mid-postmenopause stage of a naturally occurring menopause^1^ and responded to demographic questions on age, ethnicity, gender identity, and socioeconomic status.^45^

#### Focus group questions

There were 4 broad questions: (1) what helps and (2) what hinders you to be physically active during the menopause life stage, (3) how does being physically active influence your mental well-being during the menopause life stage, and (4) what could we do better/differently to support women experiencing the menopause to be physically active? (see Supplementary File 1, Supplemental Digital Content 1, http://links.lww.com/MENO/B410 for full questions). Questions 1 and 2 in the focus groups directly addressed the second research question of this paper, and some responses to question 3 also contributed. Responses to question 4 are not included in this paper and will be reported elsewhere.

### Procedure

After institutional sponsorship and ethical approval (Ref: ANIV08092022), participants received an email invitation to participate, and a link to the online participant information sheet, consent form, and the questionnaire including questions relating to menopause status, demographic characteristics, and preferred focus group time. Following consent, individuals received a MicroSoft (MS) teams link for their preferred focus group time, and a reminder was sent 2 to 3 days before the focus group. Focus groups were populated on a first come, first served basis with each participant taking part in one focus group.

Four 1-hour focus groups took place through MS teams (October 2022) and were facilitated by 3 female researchers (A.G.N, C.F., and J.R.). Two researchers (A.G.N and C.F.) are experienced qualitative researchers, educated to PhD level. One researcher (J.R.) was the research assistant, facilitating the recruitment process and liaising with participants in advance of the session. At the start of each focus group, the research team introduced themselves and briefly explained personal interest in the topic, and provided some brief background to the topic. The discussion was then focused around the 4 broad questions with care taken to encourage responses from all participants. All focus groups were recorded, and the MS teams' automated transcribing function was used to generate a transcript for each focus group. Following the focus group, participants were sent a list of resources from trusted organizations that provide support on the menopause life stage.

### Analysis

The automated transcripts were reviewed for coherence, checked against the audio recording, and modified for accuracy where needed. Initially, the data were organized into MSWord documents collating the responses to each of the questions (by J.R.). The data were analyzed thematically within an interpretive paradigm and drew from the principles of thematic analysis.^48^ Focus group responses were deductively organized into broad categories guided by COM-B relating to capability (physical and psychological), opportunity (social and physical), and motivation (automatic and reflective) by the researcher (C.F.). Within these broad categories, texts with similar meaning were clustered together to inductively identify themes, and where appropriate, subthemes. The themes and subthemes were reviewed for coherence within and between themes. Throughout the analysis process, the researcher (A.G.N.) acted as a “critical friend” to encourage discussion and reflexivity relating to decision making.^49^

## RESULTS

Twenty-four women (average age = 52.7; SD = 0.97 y; premenopause n = 12; early postmenopause n = 4; mid postmenopause n = 8) participated in the focus groups. There was a representation of the different menopause life stages across each focus group, with the exception of group 4, where there were no early postmenopausal participants. All participants were White with a gender identity that matched their assigned sex at birth. Based on SIMD, there was a spread of socioeconomic status across all 5 SIMD groupings, although there was a skew towards the areas of least deprivation with more than 50% of participants in the 2 areas of lowest deprivation.

### Influences on women’s physical activity behavior during the menopause life stage

#### Physical and psychological capability

Table 4 illustrates the 3 general themes, 10 subthemes and associated quotes related to physical capability. Participants reported that having access to hormone therapy helped them to manage symptoms of menopause and, therefore, to increase physical activity levels. However, for a small number of participants, their physical capability varied across the month due to hormone therapy, with the progesterone phase identified as being particularly troublesome.

**TABLE 4:** Influences on women’s physical activity behavior during the MLS: physical and psychological capability

Capability	General theme (and impact on PA)	Subtheme (and impact on PA)	Example quote
Physical	Hormone therapy (+/-)	Hormone therapy (+)	“…. *I’ve reduced quite a lot of this exercise that I was doing. I used to run, stopped running,…. and started HRT and I’m able to go up hills and run if I feel like it. So yeah, actually helped me and certainly keeps me as active as I am, I think”* (P32; FG4)
		Progesterone phase of hormone therapy (-)	“…*The progesterone part of it, Oh my goodness. I mean it is just sometimes I feel like I’ve been hit by something, you know, hit by a solid brick wall or something and you really have to push through it then to find the motivation to keep going and keep active*…” (P32; FG4)
	Menopausal symptoms (-)	Hot flushes, overheating (-)	“*But the other thing I get; I find the heat affects me much more with exercise*.” (P20; FG4)
		Pain (-)	“*Yeah, I mean I went for a walk today and I lasted about 30 minutes to the point I said I have to stop now because my hip joints and my knee joints and everything were so sore*.” (P2; FG1)
		Pelvic floor problems (-)	*“But I would also see um. Like my pelvic floor? Like, does that mean? Like, I used to love going like aerobic dances and aerobic classes and Zumba and all that and boxer size and stuff like that. I just couldn’t do it, especially in perimenopausal. I just couldn’t. And then I had a repair done, but our second repair done. But again, there was always that fear of leaking and stuff like that, so I didn’t really want to do that. Um.”* (P22; FG4)
		Weight gain (-)	*“So surprise surprise, I don’t want to exercise because I don’t wanna go to a swimming pool. I don’t want to wear clothes for somebody might be able to see my tummy. You know, even if I wanted to, I couldn’t do some of the things that I used to enjoy and be able to do easily.”* (P2; FG1)
		Tiredness (-)	*“I was doing that was really helpful to me was like 15 minutes of yoga just before bedtime. And that was really helping me sleep. And now I’m just going, oh, I’m too tired*…” (P33; FG4)
		Injuries (-)	*“So I think UM like you were saying there, it kind of the niggles don’t really go away. So for example I I’m a really keen runner and I’ve had a every time I run I can run regularly for* *2 months and then my calf muscle goes and at the moment it’s my hamstring and so I tend to kind of Um, so then I would maybe go swimming more rather than um rather than running. So I kind of try and do different things because parts of my body are just a little bit sore or not recovering as well.” (P13; FG4)*
		Prone to illness/injury (-)	*“I do go occasionally, but what I found was I kept getting ill every month, I’d get like a viral bug or something and I’d get really run down and I suddenly thought this doesn’t suit me.” (P29; FG3)*
		Heavy periods (-)	*“I used to go swimming* *3 times a week, but then during perimenopause that irregularity and then they are absolute a brutal mess of heavy periods. I mean, 2 weeks of constant heavy, heavy bleeding. I didn’t know when my period was coming on. I didn’t know what it would be like when it did and over about a year that swimming actually stopped because it just you couldn’t go 3 times a week because you just never knew what situation would you would be in.”* (P21; FG3)
	Variability in physical capability (+/-)		*“You know, being active does help my mental health, but if my mental health is very bad and I’m not sleeping, that is absolutely exhausting. And if you then try and push your body, you can do more harm than good. So it’s knowing. When to try and exercise and when to not do it because you’re gonna make things worse.”* (P7; FG1)
Psychological	Behavioral regulation (+)	Avoid procrastination	*“Right. I’m going to do this 54321. And then you act,”* (P21; FG3)
		Time management	*“…that was the only way it worked for me is to to get it down on paper and say it’s in the plan. Go and do it now*”, (P18; FG3)
		Pay for class in advance	*“and I pay for my yoga on a monthly basis before classes, so I’m gutted if I miss a class cause that means I’ve lost £10*” (P29; FG3)
		Making plans with other people	*“I would say a friend. Just having someone saying on Monday, we’re going to meet and we’re gonna do X. And sometimes you then are forced to do otherwise life takes over and you think ohh. I’ve got so much to do and I, you know I can’t take that time out. But the fact you’ve made a commitment to someone forces you to do it.”* (P1; FG1)
	Decline in mental health (-)	-	*“so like my brain just doesn’t function”* (P6; FG2) “*it’s definitely my mood has definitely flattened and I’m not as interested if anything is and whatever and that’s how I know that there’s I’m definitely experiencing something because it’s just not me.2* ” (P19; FG4) *“And I looked over and just like Oh my God, this is menopause, this isn’t real. This is actually menopause anxiety. And then I started to see it as its own entity that suddenly I would get really anxious about things that had never crossed my mind to get anxious about before, and one of those things was perhaps going into situations with other people going into exercise situations, going out and walk, and some people have already talked about looking like all you’re overweight or you’re huffing and puffing or and I think it’s easy once that anxiety it’s present to then allow it to start talking to you.”* (P21; FG3)

HRT, hormone replacement therapy; MLS, menopause life stage.

Many participants spoke in detail about a wide range of menopause symptoms that were barriers on their physical capability to be active. These symptoms were clustered into 8 subthemes and included hot flushes, pain due to painful joints, leg cramps and stiffness, pelvic floor problems, weight gain, tiredness including reduced energy, poor sleep and apathy, prevalence of injuries including recurrence of old injuries, being more susceptible to injuries or illness, and heavy periods. There was also discussion around how physical capability could vary, and this was clearly linked to mental well-being. Some participants were cautious about overdoing it, aware they might feel good at the time, but it could make things worse in the long run.

Table 4 also presents the 2 general themes and example quotes relating to psychological capability. One theme was labeled behavioral regulation and had 4 subthemes. This theme captured participants’ comments about avoiding procrastination and managing their time and resources (eg, pay for class in advance) carefully to ensure their intentions and/or goals were carried out. Making both short-term (eg, go for a walk) or long-term (eg, train together for a goal) plans with other people was perceived as an important way to ensure an activity happened.

Many participants spoke about how changes in their mental health during the MLS impacted their psychological capability to be active. Low or “flattening” of mood, feelings of being overwhelmed, feelings of stress, anxiety, negative thoughts, feelings of brain fog and more general discussion around mental health were all discussed.

#### Social and physical opportunity

As illustrated in Table 5, there were 8 general themes around social and physical opportunities relating to physical activity during the MLS. In relation to social opportunity, participants spoke about how the support from other people including family or friends helped them to stay active during the MLS. This support could be practically oriented to support coparticipation and emotionally oriented providing women with an opportunity to socially connect, discuss their lives, including menopause or providing support and encouragement to be active. For some participants, there were clearly social norms around physical activity within their networks that continued into the MLS and encouraged activity. Similarly, participants also discussed the wider context around menopause, with a general perception that social norms around menopause are shifting, with menopause discussed more openly. This normalizing of menopause was also evident within physical activity. While social networks could be supportive, some women also talked about how managing multiple responsibilities at this point in their lives could lead to them “feeling squeezed” and negatively impacting on the time available.

**TABLE 5:** Influences on women’s physical activity behavior during the MLS: social and physical opportunity

Opportunity	General theme (and impact on PA)	Subtheme (and impact on PA)	Example quote
Social	The support from other people (+)	Coparticipation	*“I’ve got a made a couple of groups of friends that I do physical exercise with or have done in the past that has made it fun. So rather than sort of being forced to, you know…”* (P2; FG1)
		Emotional support	*“Being part of a team, being part of a group of women. Where everybody’s got your back, so to speak.”* (P12; FG2) *“But I also think what I miss most is the social interaction with others. You know, if there’s classes or when there were things on that I felt I was able to go to and you felt better for being among others as well. And so, you know, it was good for me socially.” (*P26; FG4)
	Social norms around activity (+)		*“I feel like if I don’t get out for a walk. My whole family like this, all the women in my family, like we have to be walked once a day.”* (P33; FG4)
	Normalizing menopause (+)		*“Yeah. I mean it’s quite interesting because I feel like for the first time in my life I’m really fashionable because menopause is talked about all the time. And I think that’s a really good thing that, you know, there’s menopause at work or there’s menopause jog Scotland groups, there’s menopause cycling groups and it just feels like I have come to this stage of life at the right time, and it would have been different if I got there 10 years ago.”* (P13; FG4)
	Multiple responsibilities (-)		*“And I suppose one of the other thing that made hinder women even managing some time for themselves is maybe the life stage that they experience the menopause at you know some of them in their 40s still have quite young children or you have teenagers and then you have aging parents….”* (P18; FG3)
Physical	Having opportunities available (+/-)	Physical activity opportunities (+/-)	*“Woman, let’s face it, 51% of the population experiences menopause of some kind. Yet there’s no gyms, no classes, no Particular activities that are Tailored to the needs of menopausal perimenopausal or postmenopausal women, and I do find that frustrating.”* (P21; FG3)
		Supportive environment (+)	*“I’m tired, but I think gardening really been my savior. You know, just getting outside and spending time outside and. And as you can see, I’ve got quite a big garden, so it sort of keeps that side and getting out for walks and things as well.”* (P14; FG1)
	Having access to hormone therapy/supportive health care professionals (+/-)		*“The only thing that changed it for me was a discussion with the GP* *and the GP* *saying “Do you wanna try HRT* *” and within 3 weeks I felt like a different person. It was literally like a miracle. Felt like the energy was back. My motivation was back. So I was still trying to do all the exercise, but it was the hormonal side that was really dragging me down.”* (P18; FG3) *“Had I known that all of these different things all add up to menopause, then I would have been to see my GP* *a lot quicker. And especially had I known that I would have been fighting for over a year to actually get them to take me seriously and prescribe me HRT.”* (P23; FG2)
	Having a dog (+)		*“And the main thing that I did do, I’ve got a dog. So my dog is a rescue. We don’t know our history, but Likes to go out 4 times a day and um Oh my God. And she hunts. So I’m around and I could be out in the morning. I’m out for 40 minutes before I start work, which has been really good actually for. Get moving forward and amount of lunch and about every time. And then he does the evening walk. So hey, but I have to say that’s really helped because we run up the field sometimes and then I wouldn’t have run last year.”* (P19; FG4)
	Lack of time (-)		*“And the first thing really it was that the kids came first. The house came first work, and then if I if I had enough energy left, then activity. But to be honest, I found that really quite tricky.”* (P1; FG1)

GP, general practitioner; HRT, hormone replacement therapy; MLS, menopause life stage.

In relation to physical opportunity, participants spoke about how having opportunities available supported them to be active during the menopause life stage. Participants discussed the benefits of opportunities that were “really close,” local, or easily accessible including online opportunities, local gym classes, Jog Scotland groups, walking group with friends, etc. In contrast, some participants highlighted a lack of exercise classes in their local area that were appropriate or suitable during the menopause. Some participants also mentioned they lived in a supportive environment such as having a garden, being near woodlands, or the importance of benches to rest when out for a walk.  Being able to access a supportive family doctor or health care team, and having the opportunity to try hormone therapy, were mentioned as helping people to stay active. Not all women had the same positive experience, with some having less supportive health care provision where it was difficult to get hormone therapy, and to get the hormone therapy dose correct, and others unaware that they could be seeking support. Many of the participants spoke about how having a dog provided an opportunity to be active and including walks into their day was something that had to be done to exercise the dog. Linked to the multiple responsibilities theme in social opportunity, for some participants finding time to be active at this stage in their lives was challenging due to caring for other people or juggling other responsibilities.

#### Reflective and automatic motivation

As illustrated in Table 6, there were 7 general themes around reflective and automatic motivation, relating to physical activity during the menopause life stage. Participants talked about a range of beliefs about the consequences of being physically active primarily acting as facilitators, but not all. Many of the participants were aware that being physically active made them feel better and eased mental health symptoms of the menopause such as low mood, anxiety or stress. Participants explicitly spoke about the positive effect that being active had on their mental well-being, and how they wanted to use this to motivate themselves to be active again. In contrast to those individuals who felt that being active made them feel better, one participant spoke about how she did not feel this way and struggled to understand why they found it beneficial. Some participants were conscious of the aging process and not getting any younger, and their beliefs that being active would help them age well were motivating. Some participants were aware of the importance of self-care and looking after themselves during the menopause life stage, listening to their body and not overdoing things. Related to beliefs about the consequences of being active, participants also spoke about their belief that being outdoors made them feel better. For some, although not all, being outdoors was related to being active. For some, motivation was boosted by having a goal and something to train for, giving them a focus to stay active and intention to keep going. Participants also spoke about their beliefs in their capability to be active. For example, several highlighted that they believed they were less capable at different times of the day. For some participants, motivation was also influenced by low confidence in relation to physical activity that was present before the menopause life stage and was an ongoing issue. Overall, there was an awareness among the participants that they lacked motivation to be active as a result of the menopause life stage. Linked with the findings reported above, some participants discussed how motivation to be active had declined due to a fall in energy levels/tiredness, with challenges of caring responsibilities, work and home life depleting energy levels. Fluctuations in energy levels at different times of the month also affected motivation to be active, as was a general lack of enthusiasm/flattening of mood and apathy. There was a sense of confusion in some participants why motivating yourself to be active was so challenging when you get such clear benefits from it.

**TABLE 6:** Influences on women’s physical activity behavior during the MLS: reflective and automatic motivation

Motivation	General theme (and impact on PA)	Subtheme (and impact on PA)	Example quote
Reflective	Beliefs about the consequences of being active (+/-)	Aware being active made them feel better (+)	“*Umm, I’ve always suffered from anxiety and I really because of life circumstances in the last few years. I don’t know what I can attribute to menopause and what’s just natural and stresses and anxieties. But the motivation to exercise because of the impact on my anxiety, the positive impact is absolutely massive.”* (P10; FG2)
		Beliefs about positive consequences for mental well-being (+)	*“I always knew it would be better once I once I came back and I would always regret it if I hadn’t done it.”* (P18; FG3)
		Lack of benefits from being active (-)	*“I have never found physical activity to help me in any way at all except to make me hot and sweaty. I don’t get this buzz that everyone talks about, you know, all the endorphins after it and you know, never had it. So I’m feeling a bit kind of odd listening to people saying how, how beneficial it can be. Just just to say that, yeah, there is a different. I have different viewpoint here.”* (P16; FG3)
		Beliefs about the positive consequences for aging (+)	*“… I want to live until I’m about 120 and I want to be walking and you know, I want to be well the whole way through it. So that’s what motivates me the most….”* (P20; FG4)
		Self-Care (+/-)	*“I kind of think you know, you’ve gotta listen to your own body and what your body is capable and and mine at the moment isn’t capable of that. And maybe when I squirt out the other end, then that can become just like them. But the minute I can’t.”* (P1; FG1)
	Beliefs about the consequences of being outside (+)		*“And I so I do find that if I don’t get outside and get some fresh air then you know, my anxiety can really go through the roof and it is, it really does strike me that if I get outside just for 10 minutes and get some fresh air, walk around the block even it makes such a difference to just my A general well-being, but also mental well-being, definitely.”* (P32; FG4)
	Intentions and Goals to be active		*“And I have to like when the other person was saying, like I did the kilt walk and the moonwalk, but I had to do, I had to set that as a target to do it or I would never have done it.”* (P19; FG4)
	Beliefs about capabilities	Influence of time of day (+/-)	*“But I mean things like, I used to love running in the mornings, but now I can’t even be bothered to get myself out of bed before 9:00 o’clock.”* (P20; FG4)
		Lack of confidence to be active (-)	*“…I’m convinced when I run past people, they go look at that fat cow, look how much she’s sweating and stuff like that. So I’ve got absolutely no confidence exercise-wise, never have as a child.”* (P16; FG3)
	Lack of motivation to be active (-)		*“You have to fight. You definitely have to find something that works for you, I think. And I think for all of us, that’s all something different. But there is something that I think menopause really affects your ability to motivate yourself in a way you once did. And I said that I don’t know whether it’s because you don’t feel in control of your body anymore. Whereas when I was in younger, I felt it quite in control of my body. But with menopause, I didn’t feel in control of my body.”* (P11; FG3)
Automatic	Physical Activity as a habit or normal routine (+)		*“And I have played netball all my life since I was 7 and I’m now almost 52.”* (P12; FG2)
	Emotional frustration (-)		*“Sometimes I find it frustrating. Because you see, you know, some of the celebrity menopause superstars doing these amazing workouts and managing to do all this stuff and you think how come they can still manage to do? All that sport and and I I can’t*.” (P1; FG1)

MLS, menopause life stage; PA, physical activity.

In relation to automatic motivation, some participants spoke about how being active was a habit or a normal part of their routine to walk, walk the dog or engage in active transport, and this supported them in being active. A sense of frustration was mentioned by one participant around celebrity role models and their role in the menopause life stage around physical activity.

## DISCUSSION

Growing evidence highlights the benefits of physical activity during the menopause life stage.^17^,^18^,^19^,^20^,^21^,^22^,^23^ The aim of this study was to increase understanding of the physical activity behavior of UK-based women, the relationship with menopausal symptoms, and the theory-informed influences on this behavior. These findings can be used to inform how best to support women to be active during the menopause.

### Phase 1

The sample in phase 1 represented both perimenopausal and early-postmenopausal and mid-postmenopausal women, who were predominantly White and identified with their gender assigned at birth. Among this self-selected sample of predominantly White perimenopausal, early-postmenopausal and mid-postmenopausal women, 75% reported meeting the MVPA guidelines and 48% met both MVPA and muscle strength guidelines. This is similar to Scottish nationally representative data^27^ which indicates that the percentage of those achieving the recommendations for MVPA levels was similar (ie, 75% vs 74% and 73% of women aged 35 to 44 and 45 to 54), but more of the current sample undertook both MVPA and muscle strengthening compared with national representative data (ie, 48% vs 40% and 34% of women aged 35 to 44 and 45 to 54). It should therefore be noted that the participants were somewhat more physically active than the typical population, likely reflecting a sampling bias where those interested in the topic of physical activity were more likely to encounter the recruitment materials and also volunteer to participate. Nevertheless, more than half of participants indicated that their physical activity levels had declined during the menopause life stage, which is higher than the 30% reported previously,^44^ and 75% were interested in becoming more active.

Consistent with previous reports,^44,50^ the participants reported that menopausal symptoms were common, with 12 of the 14 identified symptoms experienced by more than 50% of the sample. The findings indicated a mixed picture on the relationship between menopausal symptoms and physical activity. There was no significant difference on the proportion meeting the MVPA guidelines between those women who did or did not experience individual symptoms. Nevertheless, it is notable that for the symptoms, “muscle ache and joint pains,” “difficulty sleeping (not due to night sweats),” and “brain fog,” the proportion meeting the guidelines was ~10% higher for those not experiencing the symptom, albeit large CIs were evident. Interestingly, a reverse nonsignificant relationship was evident for the symptom “difficulty sleeping (due to night sweats),” where the proportion meeting the guidelines was 9.5% higher for those experiencing this symptom. The relationship between vasomotor symptoms and PA is complex with inconsistent findings not yet untangling the circumstances in which exercise may be a trigger or treatment.^23,51^ The lack of significant differences evident in these data may indicate a lack of relationship between physical activity and menopausal symptoms, or may be due to our stringent threshold to account for multiple comparisons, or limitations in the validity of the self-report physical activity measure.

In contrast to these nonsignificant findings, many of the participants reported that the common symptoms of menopause had an adverse effect on their likelihood to engage in physical activity, consistent with previous studies.^18,29,44^ Specifically, for 10 out of the 14 symptoms, more than 50% of respondents indicated the symptom had a negative impact on their likelihood to engage in physical activity. “Changes to mood,” and “difficulty sleeping (not due to night sweats),” and “muscle aches and joint pains” had the most detrimental impact with around 70% of respondents indicating these symptoms resulted in them being a lot less likely or less likely to engage in physical activity. Although further research is required, collectively these findings highlight that “difficulty sleeping (not due to night sweats),” and “muscle aches and joint pains” could be key symptoms to consider further in relation to physical activity.

### Phase 2

The findings from phase 2 of this study complemented phase 1 by providing a deeper and novel understanding of the factors influential on the physical activity behavior of a sample of 24 UK-based women during the menopause life stage. Utilizing the COM-B model,^41^ it was evident that a broad range of factors influenced behavior across capability, opportunity and motivation. The largest theme was physical capability, and consistent with some of the findings in phase 1 and previous research,^44^ the women reported a wide range of menopausal symptoms that negatively impacted capability to be physically active. The predominance of the influence of physical capability is consistent with the findings of a recent systematic review of the barriers and facilitators to being physically active^29^ during the menopause that highlighted physical health, including menopausal symptoms, as a prominent barrier. There is a need to support women to best manage menopausal symptoms to be physically active, and subsequently benefit from the positive impact of physical activity on menopausal symptoms.^20^,^21^,^22^,^23^ As reported by the participants, having access to hormone therapy facilitated being active and, for some women, this may be appropriate.

Linked with the physical capability barriers, many participants were also aware of a decline in psychological capability with changes to mental health making it harder to engage in physical activity. Encouragingly, within this theme, participants reported a range of behavioral regulation strategies that helped them to be active including managing time and resources carefully to ensure intentions to be active were carried out. There is consistent evidence on the value of such planning strategies to support physical activity behavior,^52^ and developing resources to support women to do this most effectively could be valuable.

A further prevalent facilitating theme within the social opportunity category was the support of other people, providing both practical participation and emotional support in the short and longer term. These findings reinforce the conclusions from a systematic review by Casey et al^29^ that fostering social support is important to promote physical activity engagement during the menopause, and creating opportunities to be active with others may be beneficial. Social norms around activity and normalization of discussion around menopause were also facilitators, and the positive effects of opportunities to talk about menopausal symptoms and engage with others going through similar experiences has previously been reported.^32^ For some participants, a busy social environment at this life stage meant they had multiple responsibilities to manage, resulting in a lack of time to be physically active. The impact of competing demands on physical activity for women at this life stage has been noted previously,^40^ and, therefore, physical activity opportunities that are flexible and can “fit in” may be most beneficial.

Physical opportunities that facilitated activity related to having access to appropriate local physical activity opportunities, along with access to supportive health care staff and hormone therapy (if needed). However, consistent with previous findings,^8^ the participants reported varied experiences regarding the quality of health care support. In line with previous research, having a dog in the house facilitated regular activity.^40^ Barriers included a lack of time, a lack of suitable opportunities to be active, as well as a lack of support from health care professionals.^39^ Ultimately, all women should have access to appropriate health care support during the menopause life-stage, with inequitable access to services a potential mechanism to further contribute to prevalence of chronic conditions associated with the menopause life stage.^53^

Encouragingly, in relation to reflective motivation, activity was facilitated through positive beliefs about the consequences of being active and also being outside. Similar to previous research, the positive effects of being active on mental well-being were acknowledged,^29,32,39,40^ and a desire to stay active and healthy with increasing age^38^ was motivating. However, there was an awareness among the participants that motivation to be active had decreased as a result of the menopause life stage, with low levels of confidence, low energy, and multiple demands on the participants contributing to this decrease. Other studies have also noted the prevalence of lack of motivation in this population.^29,39^ For some participants in this study, there was a sense of confusion why motivating oneself to be active was so hard when there were clear benefits from it. Acknowledging and accepting this contradiction and supporting women to identify achievable opportunities may help women be active. Finally, in relation to automatic motivation, and as noted in other studies,^29^ when physical activity was a habit or part of normal routine then this facilitated being active.

From this understanding of menopausal women’s lived experiences of physical activity, it is likely that a range of options would work best, with activities that can be flexible and “fit in” with busy lives most likely to be feasible. Walking is one such activity, and previous research has also highlighted preferences for walking during menopause.^35^ Further, highlighting the benefits of physical activity during the menopause life stage, and equipping women with behavioral regulation strategies to enact good intentions, is recommended to facilitate engagement in physical activity. Future research should focus on codeveloping acceptable interventions that can incorporate these characteristics to help enhance current and future health and well-being of women in the menopause life stage.

### Strengths and limitations

Adopting a mixed methods approach, utilizing a theoretical framework, is a strength to this study as it provides a fuller picture of both the what and also the why of physical activity behavior in menopause. In addition, precise use of menopause nomenclature^4^ is a strength of this study ensuring the focus is on participants who are in the menopause life stage that is most impacted by symptoms.^1^ It is a strength of this study that the focus was on general physical activity rather than responses to a specific physical activity program or intervention as this enhances the applicability of the findings and adds to the limited evidence base.

However, there are limitations that should be acknowledged and considered in the interpretation of the quantitative findings based on the included sample. Our recruitment and sampling strategies mean that the sample is not representative of the wider population, but nevertheless, the sample was not substantially more active in terms of MVPA than national representative data. A lack of cultural diversity in the sample is a further limitation of the study, and future research should seek to specifically target for recruitment a range of ethnic groups to explore the influence of cultural differences on physical activity experiences during the menopause transition.^18^ Further, the high attrition rate for completing the questionnaire is likely due to our high threshold for consent requiring participants to formally “submit” their responses, but may nevertheless have resulted in a skewed sample. Furthermore, the relatively low sample size is a limiting factor in detecting significant differences between groups, once adjustments for multiple comparisons have been made. The use of self-report physical activity measurement can raise concerns regarding reliability and validity, so future research adopting device-based measurement tools would be valuable to build a more accurate understanding of behavior.^54^ A further limitation is the cross-sectional design of the questionnaire study, which, as noted in the introduction, limits the ability to untangle the direction of effect between menopausal symptoms and physical activity levels. Future research adopting longitudinal designs are essential to examine the causal relationships between physical activity and symptoms across the menopause life stage.

## CONCLUSION

The findings of this study provide a novel insight into the physical activity behavior of UK-based women in the menopause life stage. Consistent with previous research,^29^ it is evident that there are multiple factors influencing physical activity behavior, with variation between women. Nevertheless, there is a consistent theme that the symptoms of menopause can be a barrier and can reduce the likelihood of women being physically active. Given the benefits of physical activity for health and managing symptoms,^20^,^21^,^22^,^23^ identifying how best to support women during the menopause life stage to be active is a priority.
